# Bone Health in Premenopausal Women with Coeliac Disease: An Observational Study

**DOI:** 10.3390/nu16142178

**Published:** 2024-07-09

**Authors:** Katie Schraders, Jane Coad, Marlena Kruger

**Affiliations:** 1 School of Food Technology and Natural Sciences, College of Sciences, Massey University, Palmerston North 4442, New Zealand; j.coad@massey.ac.nz; 2 School of Health Sciences, College of Health, Massey University, Palmerston North 4442, New Zealand; m.c.kruger@massey.ac.nz

**Keywords:** coeliac disease, CD, gluten free diet, GFD, quantitative ultrasound, QUS, dual X-ray absorptiometry, DXA, bone mineral density, BMD, bone mineral content, BMC, young women, calcium, protein

## Abstract

Low bone mineral density (BMD) is common in adults with coeliac disease (CD), even in individuals adhering to a gluten-free diet (GFD). Women are more likely to have low BMD and have an increased risk of osteoporosis, so women with pre-existing low BMD related to CD are at an even higher risk. BMD assessed by dual X-ray absorptiometry (DXA) and bone quality assessed through quantitative ultrasound (QUS) were investigated in 31 premenopausal women with CD consuming a GFD, and 39 matched healthy controls from the Lower North Island, New Zealand. In addition, bone metabolism and nutrient status were assessed, and four-day diet diaries were used to estimate nutrient intake. No statistically significant differences were found in BMD assessed by DXA between the two groups at the hip, lumbar spine or forearm. However, the parameters measured by the QUS were significantly lower in CD participants. Dietary data indicated significantly lower intakes of energy, dietary fibre, magnesium and phosphorus in women with CD, likely as a result of a reduced intake of wholegrain foods, and suggested that both groups had inadequate intake of calcium. No significant differences were demonstrated in biochemical parameters. BMD and bone biomarkers indicated no differences between coeliac and healthy women in New Zealand. However, these findings suggest that QUS may be more sensitive for the coeliac population, due to the disease’s affect on the trabecular bone, and warrant further research.

## 1. Introduction

Coeliac disease (CD) is an immune-mediated disease that is triggered in response to the consumption of gluten in the diet [1], in individuals who are genetically predisposed and express the HLA-DQ2 and/or HLA-DQ8 genes [2]. Diagnosis of the disease is confirmed by the presence of autoantibodies (anti-tissue transglutaminase, tTG and anti-endomysial antibodies, EMA), and evidence of intestinal damage assessed through endoscopic biopsy [2]. In addition to intestinal symptoms, many individuals experience extra-intestinal symptoms resulting from both inflammation and the malabsorption of essential nutrients [3]. The malabsorption of nutrients as a result of inflammation and villous atrophy is common, compromising the absorption of calcium, protein and other essential nutrients [4]. As a result, bone mass is often affected [5]. The removal of gluten from the diet results in the resolution of gut damage in most individuals with CD, with gut inflammation and nutrient absorption dramatically improved [6]. However, even with rigorous compliance to a gluten-free diet (GFD) after diagnosis, a full recovery of bone density is not always achieved in a significant proportion of individuals [6,7]. Low bone density is therefore a frequent finding in people with CD at the time of diagnosis, which may persist [8].

Despite New Zealand having one of the highest known prevalence of CD in the world at 1.2% [9], with many still believed to be undiagnosed [10], very little research has been conducted investigating bone health in this population group. Previous research studies have assessed the prevalence of CD in individuals who have undergone a DXA scan for other reasons [11]. This approach assesses patients who have been referred for DXA and does not accurately report on the prevalence of low bone density within the coeliac population. Additionally, although the Coeliac Society of New Zealand recommends all adult patients diagnosed with CD speak to a health professional about a DXA scan [12], research suggests that practitioners in New Zealand do not routinely refer CD patients for BMD assessment [13]. Osteoporosis more commonly affects women [14], with menopause and the associated decline in oestrogen resulting in a sharp increase in the rate of loss in bone density [15]. As both the onset of menopause and the rate of loss in bone density can vary significantly between women, an assessment of premenopausal women offers the opportunity to assess the risk of developing osteoporosis in later life and to potentially offer health advice to limit the further loss of bone. As the prevalence of both CD and osteoporosis are greater in women than in men [16], women with CD have a heightened health burden associated with osteoporosis. This study, therefore, aimed to investigate bone density, and the factors affecting bones, in premenopausal women with CD compared to a matched healthy control group. 

## 2. Materials and Methods

### 2.1. Study Design

This cross-sectional study was undertaken at Massey University, Palmerston North campus. Participants visited the Human Nutrition Research Unit (HNRU), at Massey University, after fasting overnight but remaining hydrated, where they had blood samples drawn, were provided with breakfast, completed questionnaires about their medical history and diet, and had BMD and bone quality assessed by DXA and QUS, respectively. 

### 2.2. Participants

Participants were recruited through the use of advertising around the university campus and through social media, with advertising targeted to people consuming a gluten free diet. Participants were matched based predominantly on age and body mass index (BMI) (calculated from self-reported weight and height) reported in their responses to the pre-enrolment screening questionnaire. In addition, participants were asked about their self-identified ethnicity because ethnicity can affect BMD [17].

The exclusion criteria included a diagnosis of any other known condition that affects bones or nutrient absorption, such as osteogenesis imperfecta, uncontrolled thyroid disease, chronic renal disease, inflammatory bowel disease, clinically significant liver disease and a diagnosis of juvenile rheumatoid arthritis, as well as the use of oral corticosteroids for 3 months or more. Coeliac participants were also excluded if they had not received a medical diagnosis confirmed through gastroenterology. Coeliac participants also needed to be diagnosed at least a year before participation and to report strict adherence to a GFD at the time of their visit. All participants were fully informed about the requirements of the study, both prior to and at the start of their visit to the Human Nutrition Research Unit, and gave written consent. The study was registered through Australian New Zealand Clinical Trials Registry (ANZCTR), Trial ID ACTRN12619001542189, and was approved by the Massey University Human Ethics Committee (Southern A), Reference Number SOA 18/73. 

### 2.3. Dietary Analysis

Participants were asked to complete a four-day estimated food record prior to their visit to the research unit. Participants were asked to record all food or beverages they consumed each day, on three weekdays and one weekend day. They were advised to be as accurate as possible, to use household measures to estimate intake, and to provide any recipes used, or images or food packaging where appropriate. In addition, participants were asked to provide a separate list of all supplements currently being consumed, including the brand, quantity consumed and when these were taken. All food records were reviewed with participants at the time of their visit to the Human Nutrition Research Unit to address entry errors or missing values. 

Dietary data were analysed using the New Zealand database available through FoodWorks 10 Professional (Xyris Software Pty, Brisbane, Australia). In addition, Australian and American databases and NZ Food Composition Tables were utilised where suitable options were unavailable through FoodWorks. The diets of participants were compared for the average intake of nutrients which are commonly lacking (e.g., carbohydrate, dietary fibre, calcium, iron, phosphorous, magnesium and zinc) or consumed in excess (e.g., fat and sodium) in the GFD [18].

The Goldberg cut-offs were used to assess the validity of the reporting of participants’ dietary intake [19,20]. A physical activity level of 1.55 [20] was used to estimate under- and over-reporters. BMR was determined from weight, height and age using the Mifflin equations [21], with dietary data excluded where the EI:BMR ratio was outside of the 95% confidence interval (1.00–2.40) [20]. 

### 2.4. Anthropometric Measures

Participants’ height was measured to the nearest 0.1 cm using a wall-mounted stadiometer (Seca Medical Measuring Systems, Chino, CA, USA), with weight measured to the nearest 0.1 kg using accurate electronic floor scales (Life Measurements Inc., Concord, CA, USA). BMI (weight (kg)/height (m)^2^) was calculated using these parameters. In addition, whole body composition (fat; lean tissue) was assessed using DXA Horizon A (Hologic Inc., Bedford, MA, USA).

### 2.5. Bone Densitometry

DXA scans of the whole body, lumbar spine (L1–L4), left proximal femur (femur neck, Ward’s triangle and trochanter) and left forearm (distal 1/3), as well as total body composition, were carried out by a qualified operator, using a Hologic Horizon series, fan beam X-ray Bone Densitometer, model A (Hologic Inc., Bedford, MA, USA), to determine bone mineral content (BMC, grams), bone mineral density (BMD, grams/centimetre^2^) and z-scores for each participant. The calibration of the DXA was completed daily with a spine phantom, and precision determined using the coefficient of variation of 0.45–0.54% for all measures, according to the manufacturer’s instructions.

As participants in this study were premenopausal, z-scores were used to assess risk; these are more appropriate than t-scores due to being age-matched [22]. Although commonly reported in other research studies, the International Society for Clinical Densitometry (ISCD) cautions that the diagnosis of osteopenia and/or osteoporosis is not appropriate in premenopausal women [23]; z-scores are used instead to classify whether bone density was within the expected range for age (z-score > −2.0) or below expected range for age (z-score < −2.0) [24]. For premenopausal women, additional methods of assessing fracture risk, such as Vertebral Fracture Assessment, are not recommended [23]. 

### 2.6. Bone Quality

In addition to the assessment of BMD through DXA, bone quality was assessed through Quantitative Ultrasound, as this cheap and minimally invasive assessment tool has been used to assess bone quality in this type of subject group [25,26], although its use is primarily in children [27,28,29]. 

An Achilles QUS ultrasonometer (Lunar Achilles Insight, GE Lunar Corporation Inc., Madison, WI, USA) was used to establish bone quality in the calcaneus (heel) of the non-dominant leg (to minimise variability). Calibration was completed on the morning of each visit according to the manufacturer’s instructions. Broadband ultrasound attenuation (BUA) and speed of sound (SOS) were measured, with z-scores and stiffness index (SI) calculated to assess whether each participant’s bone parameters were within the expected range for age (z-score > −1.0) [30].

### 2.7. Blood Parameters

Fasted venous blood samples were collected by a qualified phlebotomist between 8:30 and 9:30 a.m. using sterile flashback vacutainer needles; the timing allowed for minimal variation in bone marker results as C-terminal telopeptide of type I collagen (CTX-I) has a circadian rhythm [31]. Serum samples were allowed to coagulate before being centrifuged.

Serum samples were used to measure 25-hydroxyvitamin D (vitamin D). In addition, markers of CD, tissue transglutaminase (tTG) and endomysial antibodies (EMA), were analysed to assess adherence to a GFD in coeliac participants and to exclude the possibility of undiagnosed CD in the healthy control group. 

Plasma samples were collected in vacutainers containing EDTA anticoagulant to measure CTX-I and placed on ice to await processing. Additional plasma was collected in lithium-heparin containing vacutainers for the analysis of serum calcium (calcium), high sensitivity C-reactive protein (hsCRP), B12 and folate.

All samples were centrifuged at 3000× *g* for 10 min at 4 °C within 2 h, after which they were aliquoted and frozen before being stored at −80 °C to await further processing at the end of the study. At the conclusion of the study, collected samples were couriered on dry ice to Canterbury Health Laboratories, Christchurch, New Zealand for analysis. 

Trained staff also collected a capillary blood sample from the finger using a lancet to measure whole blood haemoglobin concentration as a proxy for iron status using a point-of-care Hemocue Hb 201+ system (HemoCue, Ängelholm, Sweden). Prior to this procedure, participants were asked to wash their hands with warm water to remove any dirt and promote adequate blood flow in capillaries; the finger used for collection was also wiped with an alcohol wipe to reduce any further risk.

### 2.8. Physical Activity

Physical activity was assessed using the internationally validated Global Physical Activity Questionnaire (GPAQ) developed by the World Health Organization (WHO) [32]. The questionnaire required participants to answer 16 questions regarding activity at work, travel to and from places, recreational activity and sedentary behaviour. Responses were then coded and used to produce Metabolic Equivalents of Task (METs), used for estimating metabolic expenditure [32]. 

### 2.9. Questionnaires

Questionnaires were administered online using Qualtrics software 12/2019 (Qualtrics, Provo, UT, USA). Each participant’s eligibility was assessed through the screening questionnaire, which was completed at home and clarified as required by an email from the research team to confirm eligibility. At the visit, participants were asked to complete a questionnaire regarding their health. This included questions about their diagnosis, symptoms (for participants with CD) and diet.

### 2.10. Statistical Analysis

Statistical analysis was carried out using Statistical Analysis Software (SAS) (Version 9.4) (SAS Institute Inc., Cary, NC, USA). The Shapiro–Wilkes test was used to test for normal distribution, with normally distributed results reported as mean ± standard deviation (SD) and non-parametric data presented as median with 25th and 75th percentile. Comparisons between the two participant groups were carried out using the Tukey–Kramer Method and associations between groups were assessed using Fisher’s Exact Test. In addition, relationships between QUS and DXA were assessed using univariate linear regression, with the Fisher-Z transformation used to compare correlations. Statistical significance was set at *p* < 0.05.

G* Power 3 software [33] was used to calculate a sample size of 39 women in each group, which was required to detect a difference in bone density between people with CD and healthy controls, with an effect size 0.41 at power 0.95 and a probability of type 1 error 0.05 [34]. 

## 3. Results

### 3.1. Participants Characteristics

One hundred and ninety-seven women aged 18–40 years were screened for participation. Healthy controls were matched to coeliac participants, with 127 women excluded due to factors ranging from the absence of a definitive diagnosis of CD, other pre-existing conditions and not adequately matching with coeliac participants. Seventy women aged 19–40 were recruited from the Manawatū and surrounding regions. This included 31 women with CD and 39 healthy controls who took part in the study between December 2019 and June 2021. 

There was no statistically significant difference between the two groups based on these parameters or body fat percentage assessed through DXA (Table 1). In addition, no differences were found in education level between participant groups.

Participants primarily reported being of European descent, with 77.4% (24/31) of coeliac participants and 66.7% (26/39) of healthy controls self-identifying as being New Zealand European. The remaining coeliac participants and a further 20.5% (8/39) of healthy controls identified as being partially of European descent; with the remaining 12.8% healthy controls identifying as Latin American (5/39). In addition, it should be noted that Māori and Polynesian groups were under-represented in the study population, with only 9.7% (3/31) coeliac participants and 2.6% (1/39) healthy controls identifying as Māori and 2.6% (1/39) healthy controls identifying as Polynesian. Although this does not provide an accurate representation of the New Zealand population, it is not unexpected for the coeliac population, as the HLA haplotypes associated with CD are not commonly found in these ethnic groups [35].

### 3.2. Bone Density

Only 6.5% of coeliac participants (2/31) had previously had a DXA scan, compared with 12.8% healthy control participants (5/39). Six healthy controls also reported having had a previous QUS assessment, of which two were individuals who had also had a previous DXA; no coeliac participants had had a previous QUS.

The mean z-score from DXA for all sites were within the expected range for age (z-score of >−2.0) [24], both overall and within the two participant groups (Table 2). Only two participants were identified as having bone density below the expected range for age for their whole-body scans; both participants were in the coeliac group. In addition, one coeliac participant had a lumbar spine z-score lower than expected for age. No other participants had DXA results outside the expected range at any site. However, the QUS identified four participants considered at risk based on a z-score of less than −1.0 [30]. These participants were not identified as being at any risk by DXA, at any site, with all results greater than −2.0 [24]. 

An assessment of the DXA findings demonstrated a trend of lower BMD, BMC and z-score in coeliac participants at almost all sites; however, this difference was not statistically significant at the hip, spine or forearm. A significant difference between groups was demonstrated when considering only the whole-body BMD (*p* = 0.0274) and z-score (*p* = 0.0185). 

### 3.3. Evaluation of QUS

The CD group had significantly lower SI (*p* = 0.0069) and z-scores (*p* = 0.0140) compared with healthy controls (Table 3). 

A significant positive correlation was found between DXA and QUS (*p* < 0.05) in healthy controls for all points excluding the forearm (Table 4). However, a significant positive correlation was only found in the coeliac group between SI and whole-body DXA. In both groups, the low correlation coefficient indicates that a linear regression would not be a suitable fit for this relationship. No significant difference was found between correlation coefficients of the two groups. 

### 3.4. Adherence

Biological testing for tissue transglutaminase (tTG) and endomysial antibodies (EMA) was used to assess adherence to the GFD in the CD group and identify possible undiagnosed CD in the healthy control group. The results detected no evidence of undiagnosed CD and a strong adherence to the GFD; all tTG results were <3 units, indicating a negative result. One coeliac participant had a weak positive EMA result; however, their tTG, although raised, was still <3 consistent with a negative result, with hsCRP within normal range.

hsCRP was raised in six coeliac participants and two healthy controls (hsCRP > 5 mg/L indicating the presence of inflammation). However, there was no correlation with raised hsCRP and coeliac antibody results. All other hsCRP results were <5 mg/L, indicating no signs of inflammation were present.

The consumption of oats was reported by 5/31 coeliac participants. However, none of these participants had raised antibodies (tTG or EMA) or hsCRP.

### 3.5. Coeliac Participants

All coeliac participants were diagnosed by a gastroenterologist with all, but one, diagnosed through positive endoscopy. The average time since diagnosis ranged from 1 to 28.6 years, with a median time of 8.0 years (3.1, 13.5). No relationship was found between bone parameters and time since diagnosis; femoral neck BMD (*p* = 0.7692), total hip BMD (*p* = 0.9657), lumbar spine (*p* = 0.5186), distal 1/3 (*p* = 0.5511). 

### 3.6. Nutrient Intake 

The Goldberg cut-offs were used to assess validity of the reporting of participants’ dietary intake. Based on the EI:BMR ratio, dietary data from one coeliac participant and one healthy control were excluded due to being outside (under and over, respectively) the 95% confidence interval (1.00–2.40). 

The mean daily intake of calcium was 843 (±307) mg and 927 (±229) mg for the coeliac and control groups, respectively. However, only 12/25 (48.0%) coeliac participants met the Estimated Average Requirement (840 mg), while 20/31 (64.5%) of the healthy controls met this recommendation (Table 5). 

In our study, 3/30 individuals (10.0%) with CD reported lactose intolerance, compared with 2/37 (5.4%) of healthy controls. Despite this, there was no association between lactose intolerance and dietary calcium intake (*p* = 0.3409).

The average daily intake of protein for coeliac participants and healthy controls was 81.9 g and 87.5 g, respectively. There was no significant difference between these groups (*p* = 0.4208). When using weight-based guidelines to assess protein intake (0.6 g protein/kg body weight), the average intake was 1.26 g/kg for coeliac participants and 1.30 g/kg for the healthy control group. All but one participant met the EAR for protein (0.60 g/kg); with one coeliac participant having an intake of 0.57 g/kg, just below the EAR.

The dietary analysis only included food and drinks, or supplements consumed as part of a food (i.e., protein powders). Additional supplementation in the form of tablets or capsules was considered separately. Nine coeliac participants and nine healthy control participants consumed additional supplements. Of these individuals, only one healthy control and one coeliac participant reported consuming a supplement containing calcium, as part of a multi-vitamin. On average, these multi-vitamins contained insignificant levels of calcium, at roughly 50 mg each. In addition, only one coeliac participant reported consuming a vitamin D supplement, although no information regarding dosage or duration was provided. No participants reported consuming a supplement containing vitamin K.

Although no significant differences were seen in total energy intake (*p* = 0.1466), carbohydrate intake was significantly lower in the coeliac group (*p* = 0.0372). 

### 3.7. Blood Parameters

No statistically significant difference was found between coeliac participants and healthy controls in any of the blood parameters measured (Table 6). 

No participants were deficient in either B12 or folate, with only one coeliac participant having a PTH lower than the normal range. In addition, although no participants were severely vitamin D deficient, 11 participants were mildly deficient: 6 coeliac participants (21.4%) and 5 healthy controls (13.2%); however, the difference between frequency was not significant (*p* = 0.7294).

Four participants in the healthy control group had CTX-I greater than the reference value (0.75 µg/L); CTX-I was below 0.75 µg/L in all coeliac participants. The median age of participants over the reference range was 21.5 years (20.8, 22.5). 

The consideration of a reference range accounting for oral contraceptive [39] use resulted in an additional eight participants (three coeliacs and five healthy controls) being identified as having bone turnover greater than expected (>0.614 in oral contraceptive users and 0.675 in those not using them). The median age of participants over these reference ranges was 23.0 years (18.0, 25.0). 

A significant negative correlation was demonstrated between CTX-I and BMD at the femoral neck (*p* = 0.0231) and distal 1/3 (*p* = 0.0310); however, no correlation was found with total hip or lumbar spine. 

### 3.8. Physical Activity

There was no significant difference in physical activity level between groups; differences in both moderate and vigorous activity, as well as MET minutes, were not statistically significant (Table 7). Seven individuals with CD did not meet the recommendations for MET minutes compared with four healthy controls. 

## 4. Discussion

### 4.1. Bone Parameters

There were no significant differences found in bone density between coeliac women and healthy controls at the hip, lumbar spine or forearm. Although significant differences were found in whole-body BMD and z-score, it should be noted that risk identified through whole-body scans often differs from that found using more specific sites [40], and the ISCD does not recommend its use for assessment [23]; the whole-body scan was primarily included to assess body composition in this cohort. 

Previous studies have demonstrated significant differences between patients with CD when compared with matched healthy controls [6,34]. However, much of the literature assessing bone density in adults with CD has investigated bone density at the time of diagnosis [8,41] and the extent to which it improves once a GFD is adopted [42]. The evidence suggests adherence to a GFD results in initial improvements in BMD due to the resolution of gut damage; however, very little increase is seen after the first year of adhering to the diet [7,43]. In addition, there are limited studies assessing BMD in CD patients who are premenopausal, with most focusing on postmenopausal women or a wider age group including both pre- and postmenopausal women. When comparing the bone density of the CD group in our research study to the findings of other studies which have assessed BMD after adherence to the GFD for a year or more, our group had greater BMD. 

A similar study in premenopausal women with CD by Sayar et al. [44] reported a similar z-score of −0.3 at the femoral neck (−0.25 in the current study), but a much lower z-score of −0.9 at the lumbar spine (0.1 in the current study) in premenopausal women consuming a GFD for a minimum of a year. In addition, the proportion of z-scores suggestive of osteoporosis and osteopenia were greater than those found in our study, with 39.1% of participants with z-scores indicating osteopenia and 2.1% indicating osteoporosis at the femoral neck, while 65.2% had z-scores consistent with osteopenia at the lumbar spine [44]. 

In comparison after 1 year on a GFD, Szymczak [34] reported a mean z-score at the femoral neck in premenopausal coeliac women of −0.23 ± 0.98, consistent with our findings (−0.25 ± 0.87), but the z-scores at the lumbar spine (−0.14 ± 1.11), forearm (−1.23 ± 1.24) and whole body (−0.83 ± 0.92) were lower, both than in our group and those seen in their healthy control group (although these differences were not significant for femoral neck). 

The lack of significant differences between groups in the current study could indicate that both groups had low BMD. However, mean z-scores for both hip (0.19 ± 0.94) and lumbar spine (0.27 ± 0.93) from the healthy control group in this study are comparable with other research by our group, investigating bone density in healthy young women in the Manawatū region (0.19 ± 1.20 and −0.20 ± 1.13 respectively) [45]. 

There was no significant difference found in CTX-I between the two groups, suggesting no significant differences in bone turnover. There are some inconsistencies regarding the appropriate range to use for premenopausal women, with some authors suggesting different ranges dependent on the population assessed [46]. The reference range provided by Canterbury Health Laboratories [38], for example, was for all adults and does not account for the impact of oestrogen on bone turnover. In a study by Eastell et al. [47], CTX-I, measured using an automated analyser (comparable to that used in the current study) in healthy premenopausal European women, found a mean CTX-I of 0.297 µg/L, much lower than in either of our study groups (CD 0.44 ± 0.17 µg/L; healthy 0.51 ± 0.22 µg/L). However, despite the higher mean CTX I in the current study, the range in the coeliac population (0.15–0.74 µg/L) was similar to the normal range determined by Eastell’s findings (0.111–0.791 µg/L); the healthy controls in the present study had a greater upper end of the range (0.17–1.21 µg/L), indicating participants with greater bone turnover. In addition, when considering the impact of oestrogen on bone turnover, the use of oral contraceptives must be taken into account, with participants using oral contraceptives having lower bone turnover represented through lower CTX-I results [48]. When comparing the findings of the current study with that of De Papp and colleagues [39] in a similar age group, two different reference ranges in participants were proposed, dependent on whether they used oral contraceptives or not (0.08–0.614 µg/L and 0.113–0.675 µg/L respectively). The use of these reference ranges (with consideration of current oral contraceptive use) would result in an additional eight participants (three coeliacs and five healthy controls) being identified as having bone turnover greater than expected. However, of note in the current study is the average age of the participants with the greatest bone turnover. When considering those participants/individuals with CTX-I values above the reference range, either the range provided by Canterbury Health Laboratories [38] or that identified in the De Papp research [39], participants were at the younger end of the age range, with a median of 21.5 and 23.0 years, respectively. This may indicate that bone turnover is high in this population as a result of ongoing ossification because peak bone mass has not yet been achieved [49]. As the current study did not measure P1NP to assess levels of bone formation, it is not possible to draw conclusions from these results regarding whether bone resorption is high in these participants. In addition, no consideration was made for the phase of the menstrual cycle and possible cyclic variations in CTX-I in participants taking oral contraceptives [50], and insufficient information was provided by participants to assess the possible impact of the duration of contraceptive use.

#### 4.1.1. Findings from Quantitative Ultrasound

Despite no statistically significant difference between groups in bone parameters at almost all sites (excluding whole body) assessed through DXA (Table 2), statistically significant differences were observed between groups for both SI and z-score assessed through QUS (Table 3). 

In addition, three coeliac participants were identified through QUS as having a z-score of less than −1, indicating bone quality below the expected range for age. However, all three participants had z-scores within the expected range for age (>−2.0) in their DXA assessment. Two coeliac participants had whole-body z-scores of less than the expected range for age, with one coeliac lumbar spine z-score also being below the expected range for age. Despite this, all of these individuals had z-scores within the expected range for age for their QUS measurements. 

#### 4.1.2. Relationship between DXA and QUS

A statistically significant positive correlation between DXA and QUS was demonstrated in the healthy control group at all DXA sites except the forearm, consistent with findings from a previous study of women in the same region [45]. However, in the coeliac group, this relationship was less clear, with QUS only showing a significant positive correlation with whole-body DXA BMD. The weak correlation found in both groups suggests that QUS is not an appropriate method to assess the bones of an individual. In addition, these findings may indicate that the QUS of the calcaneus is not an appropriate tool to assess bones in populations with CD.

Prior research using QUS as a tool for the assessment of bones is limited in adults with CD; however, the findings of one recent study by Ballestero-Fernández et al. [25] are at odds with those of the current study, with no difference in bone quality between participants with CD and healthy controls demonstrated in premenopausal women.

The QUS used in the current research assesses the bone quality of a single site, the calcaneus, which is primarily trabecular bone and considered representative of the trabecular bone found in the hip [51]. However, the research indicates that the trabecular bone could be preferentially affected in individuals with CD who have low BMD, with Zanchetta et al. [52] reporting a 26.4% lower trabecular density in premenopausal coeliac women compared with healthy controls. It has also been demonstrated that this lower trabecular density results in reduced bone strength in the tibia and radius [41] in this age group. With abnormal trabecular bone development also identified in children with CD [53], further research is warranted to assess whether the use of QUS is appropriate for individuals with CD. 

#### 4.1.3. History of Bone Assessment

One finding of interest was the reporting of previous assessments of bones in participants. It should be noted that whereas only two individuals with CD had prior assessment of bones through DXA (6.5%), nine healthy controls (24.3%) had a previous DXA (3/39), QUS (4/39) or both (2/39). As no investigation was completed into the reason for this prior investigation of participant bone health, we cannot rule out undisclosed bone-related health concerns in our healthy control population, which may have attracted them to take part in the study. 

The low reports of previous DXA in individuals with CD in the current study is, although concerning, unsurprising based on findings from our research team [54].

### 4.2. Adherence 

Coeliac participants’ adherence to a GFD, as measured through tTG and EMA, suggested that the participants adhered well to dietary recommendations. However, it should be noted that five coeliac participants reported consuming oats in their responses to the questionnaires. The consumption of oats as part of a GFD is a contested topic in New Zealand. In most countries, wheat-free oats (oats free of contamination from wheat) are considered safe for individuals with CD [55,56], except in the small proportion (8%) who do not tolerate the avenin found in oats [57]. However, the view of experts in New Zealand is that oats are not safe for people with CD, and should be excluded from a GFD [58]. Despite this viewpoint, it should be noted that in the current study, tTG of <1 and negative EMA were detected in all participants consuming oats, consistent with no damage. 

### 4.3. Nutrient Intake

The GFD is characteristically low in dietary fibre, whilst tending to be high in fat, sugar and sodium [59]. When comparing groups, we found no significant difference between intakes (in grams) of protein, fat (both total and saturated) and sugar. However, significant differences in both carbohydrate and dietary fibre were observed, with the healthy control group consuming significantly more of both nutrients (Table 5). The lower intake of dietary fibre is consistent with previous research, which reported that the GFD is often a poor source of dietary fibre [60], primarily due to many common sources being excluded from the diet; this may be exacerbated in New Zealand, where it is advised that oats should be excluded from a GFD and that oats are labelled as containing gluten [61]. Although no significant differences were found in energy intake between groups, carbohydrate intake was significantly lower in the coeliac group (*p* = 0.0372), which was also reflected in the contribution from carbohydrate to total energy intake (*p* = 0.0267). A lower intake of carbohydrates fits with previous research findings, which reported that individuals with CD have a low intake of carbohydrates [62,63], particularly an intake of complex carbohydrates. This may also explain the differences in the intake of dietary fibre; although both groups consumed on average more than the average intake (AI), the coeliac group consumed significantly less than the healthy controls (*p* = 0.0474).

In addition to the assessment of macronutrients, mineral intake estimated from diet diaries was also compared. No statistically significant difference was found when comparing the intake of potassium, calcium, magnesium, phosphorus and zinc. This differs from previous research that identifies the GFD as having low levels of certain grains and cereal products, resulting in reduced intakes of nutrients such as magnesium and phosphorus [18]. 

Calcium intake in individuals with CD was lower than in healthy controls (841 ± 303 and 927 ± 229, respectively), but the difference was not statistically significant (*p* = 0.2271). Although fewer coeliac participants met the EAR than healthy controls (46.2% vs. 64.5%, respectively; *p* = 0.2144), the difference was not statistically significant (*p* = 0.2144). The median calcium intake of the coeliac group (819 mg) is still greater than that reported in the New Zealand Adult Nutrition Survey in 19–30-year-old women (704 mg) [64]. The reported calcium intake in coeliac participants was also greater than previous research in 18–25-year-olds in the same region (784 mg) [45]. Calcium is commonly considered a nutrient of concern in the GFD, with research suggesting that individuals with CD have an insufficient intake of calcium even if they have good adherence to a GFD, which should mean calcium absorption is not compromised [65,66]. However, the low intake of calcium is often attributed to the comorbidity of lactose intolerance and the exclusion of dairy foods. Lactose intolerance affects individuals with CD either prior to, or at time of, diagnosis. This is commonly due to a secondary insult resulting from the mucosal damage associated with gluten and a consequent reduction in lactase production [67]. Although lactose can usually be safely reintroduced once damage to the intestinal villi has healed, many individuals are incorrectly advised to permanently avoid lactose [68]. In addition, in some individuals the villi never fully recover and/or lactase production is never fully restored [69]. In the current study, lactose intolerance did not appear to influence calcium intake (*p* = 0.3409).

Due to the incomplete analysis of some foods in the food composition databases, there were insufficient data to fully examine other micronutrients of concern, such as vitamin B12, vitamin K and in particular vitamin D. However, as the diet is only a minor source of vitamin D, with most synthesised in the skin, serum levels were assessed.

The biochemical assessment of nutrient adequacy is of particular importance in this study, as some individuals with CD never experience full recovery of their intestinal villi [70]. The lack of resolution of gut damage can result in ongoing malabsorption, which may be associated with nutrient deficiencies, despite dietary data suggesting sufficient nutrients are being consumed [71]. Individuals with CD in this study had been consuming a GFD for a minimum of 1 year prior to participation, which is considered an adequate time for the villi in the gut to be restored [62], with coeliac antibodies reporting no presence of ongoing intestinal damage. Although it is not possible to conclusively confirm the resolution of gut damage without an examination of biopsy specimens, it is likely this explains why no statistical difference in blood parameters was found.

### 4.4. Strengths and Limitations

This is the first study in New Zealand which assessed bone density in young women with CD who had not previously had a DXA scan for other reasons. 

It is the researchers’ belief that additional data regarding severity of gut damage, for example through Marsh scores at the time of diagnosis, may help to provide a better indication of the true cause of low bone density in patients with CD in future research. Marsh scores provide an assessment of the extent of the damage found in the intestine during biopsy [72]. Although no correlation was found between time since diagnosis and BMD, Marsh scores would indicate the extent of the damage prior to diagnosis, which has been shown to correlate with BMD [73]. 

The analysis of the diet through FoodWorks required some assumptions to be made and substitute foods to be used, as unfortunately there were a significant number of products consumed by the participants that are unavailable in these databases, particularly gluten-free (GF) products. Although the dietary analysis provides a rough indication of intake and how the diet may differ between groups, it may not be appropriate to consider an individual’s risk factors based on this method alone, particularly given the lack of robust data about the nutrient composition of GF foods. New Zealand would benefit greatly from a GF food composition database, similar to that which has been established in Spain [74].

Additionally, it must be noted that the impact of the public health response to the SARS-CoV-2 virus may have influenced the participants’ diets over this period of data collection. Although participants were requested not to complete their food records during lockdown periods, international research suggests SARS-CoV-2 infections and strategies to mitigate against these have resulted in significant dietary alterations, which may persist after lockdowns have ended [75]. This is also further exacerbated for participants with CD, as New Zealand faced challenging supply issues for specialty food products, including GF foods [76], which persist two years later [77]. 

Furthermore, the authors acknowledge that the sample size in this study is a potential limitation. Although the sample size was powered prior to recruitment, the nature of the study taking place during a pandemic meant that it was not possible to recruit the full target numbers. Future studies investigating bone density in individuals with coeliac disease may benefit from a greater sample size.

## 5. Conclusions

Unexpectedly, we found no differences between the BMD of individuals with CD compared with healthy controls. The findings from the QUS suggest this may be a more sensitive measure for this population, due to the disease affecting trabecular bone specifically. Further research may be needed to assess the effects of CD on the trabecular bone. In addition, our findings support the international research regarding inadequacies of the GFD and raise concerns that New Zealand women may have insufficient intakes of calcium. 

## Figures and Tables

**Table 1:** Participant characteristics of premenopausal women with CD (*n* = 31) compared to healthy control group (*n* = 39).

Parameters	Coeliac Group (*n* = 31)	Healthy Control Group (*n* = 39)	*p* Value
Age (years)	27.5	25.0	0.6259
	(24.0, 36.8)	(23.8, 31.5)	
Height	167.3 ± 6.5 ^†^	168.1 ± 6.7 ^†^	0.6092
	(156.6–181.1)	(154.1–182.0)	
Weight	67.5 ± 13.9 ^†^	66.3 ± 8.4 ^†^	0.6575
	(38.0–102.4)	(45.3–88.6)	
BMI	24.2 ± 5.2 ^†^	23.5 ± 2.8 ^†^	0.4714
	(15.4–37.5)	(17.7–29.9)	
Body Fat %	37.7 ± 6.8 ^†^	35.8 ± 4.9 ^†^	0.1829
	(23.5–50.5)	(27.1–46.6)	

BMI, body mass index. Characteristics presented are from anthropometric measures taken at participants’ visit (not those that were self-reported). Data presented as mean ± standard deviation ^†^ (Range). Non-parametric data presented as median (25th percentile, 75th percentile).

**Table 2:** Description of bone parameters for premenopausal women with CD compared to healthy control group.

		Coeliac Disease	Healthy Control Group	*p* Value
**Hip**		*n* = 30	*n* = 39	
Total	BMD (g/cm^2^)	0.92 ± 0.09 ^†^	0.96 ± 0.12 ^†^	0.0765
		(0.76–1.10)	(0.77–1.16)	
	BMC	30.89 ± 4.56 ^†^	32.09 ± 5.55 ^†^	0.2423
		(21.6–40.48)	(22.15–45.19)	
	z-score	−0.13 ± 0.76 ^†^	0.19 ± 0.94 ^†^	0.0733
		(−1.5–1.4)	(−1.4–1.8)	
FN	BMD (g/cm^2^)	0.81 ± 0.10 ^†^	0.85 ± 0.12 ^†^	0.1336
		(0.67–0.99)	(0.61–1.10)	
	BMC	3.99 ± 0.56 ^†^	4.09 ± 0.69 ^†^	0.3391
		(2.92–5.05)	(2.86–5.62)	
	z-score	−0.25 ± 0.87 ^†^	0.06 ± 1.08 ^†^	0.1172
		(−1.6–1.5)	(−1.9–2.3)	
**Spine (L1–L4)**		*n* = 31	*n* = 39	
Total	BMD (g/cm^2^)	1.05 ± 0.11 ^†^	1.06 ± 0.10 ^†^	0.3816
		(0.79–1.22)	(0.88–1.25)	
	BMC	62.42 ± 9.31 ^†^	64.67 ± 9.63 ^†^	0.2242
		(39.94–83.17)	(47.44–86.69)	
	z-score	0.1 ± 1.02 ^†^	0.27 ± 0.93 ^†^	0.3295
		(−2.4–1.7)	(−1.5–1.9)	
**Whole Body**		*n* = 31	*n* = 39	
	BMD (g/cm^2^)	1.03 ± 0.06 ^†^	1.07 ± 0.07 ^†^	0.0274 *
		(0.92–1.16)	(0.95–1.22)	
	BMC	2076.33 ± 248.95 ^†^	2174.35 ± 257.18 ^†^	0.0550
		(1464.58–2558.90)	(1696.61–2762.31)	
	z-score	−0.95 ± 0.77 ^†^	−0.48 ± 0.83 ^†^	0.0185 *
		(−2.4–0.5)	(−2.0–1.3)	
**Forearm**		*n* = 27	*n* = 39	
Distal 1/3	BMD (g/cm^2^)	0.69 ± 0.04 ^†^	0.69 ± 0.03 ^†^	0.9635
		(0.63–0.77)	(0.63–0.79)	
	BMC	1.76 ± 0.23 ^†^	1.8 ± 0.17 ^†^	0.3746
		(1.35–2.21)	(1.51–2.27)	
	z-score	0.1 ± 0.71 ^†^	0.11 ± 0.58 ^†^	0.9027
		(−1.4–1.3)	(−1.0–1.7)	

Data presented as mean ± standard deviation ^†^ (Range). FN, femoral neck; BMD, bone mineral density; BMC, bone mineral content. * denotes significance; *p* value < 0.05

**Table 3:** Summary of QUS Results for premenopausal women with CD (*n* = 31) compared to healthy control group (*n* = 39).

	Coeliac Disease (*n* = 31)	Healthy Control Group (*n* = 39)	*p* Value
Stiffness Index	102.0 ± 15.6 ^†^	113.4 ± 18.4 ^†^	0.0069 *
	(77.0–135.0)	(83.0–155.0)	
z-score	0.20 ± 0.96 ^†^	0.87 ± 1.14 ^†^	0.0140 *
	(−1.40–2.20)	(−1.00–3.50)	

Data presented as mean ± standard deviation ^†^ (Range). * denotes significance; *p* value < 0.05

**Table 4:** Correlation between QUS and DXA of premenopausal women with CD (*n* = 26) and healthy control group (*n* = 39).

	Coeliac Disease (*n* = 26)			Healthy Control Group (*n* = 39)			
	Linear Regression (R^2^)	Correlation Coefficient (R)	*p* Value for Correlation	Linear Regression (R^2^)	Correlation Coefficient (R)	*p* Value for Correlation	Fisher z-Score *p* Value
SI vs. Hip BMD	0.1127	0.3357	0.0935	0.3404	0.5834	<0.0001	0.117
SI vs. LS BMD	0.071	0.2665	0.1883	0.1656	0.4069	0.0101	0.276
SI vs. FN BMD	0.1132	0.3365	0.0928	0.3554	0.5962	<0.0001	0.103
SI vs. Distal 1/3	0.0011	0.0332	0.8723	0.0859	0.2931	0.0701	0.157
SI vs. WB	0.2782	0.5274	0.0056 *	0.4465	0.6682	<0.0001	0.204

*n* = 65 due to absence of some results for particular DXA points; SI, Stiffness Index; BMD, bone mineral density; LS, lumbar spine; FN, femoral neck; WB, whole body. * denotes significance; *p* value < 0.05

**Table 5:** Nutrient intake for premenopausal women with CD (*n* = 25) compared to healthy control group (*n* = 31).

	Coeliac Disease (*n* = 25)	Healthy Control Group (*n* = 31)	*p* Value	Nutrient Reference Values (NRVs)
**Macronutrients**				
Energy Intake (kJ)	8005 ± 1648 ^†^	8584 ± 1297 ^†^	0.1466	
	(5890–12,342)	(6471–12,172)		
Protein (%EI)	16.0	16.1	0.8329	15–20 ^2^
	(14.0, 19.3)	(14.7, 18.8)		
Protein (g)	81.9 ± 29.6 ^†^	87.5 ± 22.0 ^†^	0.4208	37 (0.60 g/kg) ^1^
	(50.2–187.6)	(50.7–137.6)		
Fat (%EI)	38.9 ± 4.5 ^†^	35.8 ± 4.8 ^†^	0.0155 *	20–35% ^2^
	(27.3–47.3)	(24.9–43.5)		
Total Fat (g)	84.2 ± 19.8 ^†^	83.0 ± 16.4 ^†^	0.7876	
	(55.3–128.6)	(58.1–123)		
Saturated Fat (g)	31.3 ± 10.9 ^†^	31.3 ± 8.6 ^†^	0.9806	
	(16.2–56.1)	(15.1–47.2)		
Carbohydrate (%EI)	40.6 ± 7.0 ^†^	44.5 ± 5.7 ^†^	0.0267 *	45–65% ^2^
	(20.3–55.0)	(33.6–58.6)		
Carbohydrate (g)	193.5 ± 63.0 ^†^	224.9 ± 47.1 ^†^	0.0372 *	
	(73.8–399.5)	(151.7–327.2)		
Sugars (g)	80.0 ± 34.2 ^†^	84.7 ± 26.5 ^†^	0.5642	
	(33.9–187.1)	(31.5–141.3)		
Dietary Fibre (g)	25.4 ± 6.9 ^†^	30.3 ± 10.4 ^†^	0.0474 *	25 ^3^
	(15.2–38.0)	(12.7–51.8)		
**Micronutrients**				
Sodium (mg)	2502 ± 774 ^†^	2539 ± 775 ^†^	0.8591	460–920 ^3^
	(1241–4530)	(1342–4313)		
Potassium (mg)	2670	3185	0.4356	
	(2403, 3334)	(2728, 3514)		
Magnesium (mg)	290	385	0.0697	255 (19–30) ^1^ 265 (31–50) ^1^
	(277, 369)	(302, 418)		
Calcium (mg)	843 ± 307 ^†^	927 ± 229 ^†^	0.2476	840 ^1^
	(349–1694)	(558–1385)		
Phosphorus (mg)	1245	1405	0.0992	580 ^1^
	(1152, 1456)	(1215, 1629)		
Iron (mg)	10.77 ± 3.42 ^†^	13.53 ± 4.00 ^†^	0.0089 *	6 ^1^
	(5.33–19.05)	(6.79–21.94)		
Zinc (mg)	9.91 ± 3.15 ^†^	10.04 ± 2.53 ^†^	0.8628	12 ^1^
	(5.33–16.54)	(6.13–17.22)		

Normally distributed data presented as mean ± standard deviation ^†^ (Range). Non-parametric data presented as median (25th percentile, 75th percentile). NRVs, Nutrient Reference Values; EI, energy intake; kJ, kilojoule. ^1^ Estimated Average Requirement (EAR) [36], ^2^ Acceptable Macronutrient Distribution Range (AMDR) [36], ^3^ Average Intake (AI) [36], * denotes significance; *p* value < 0.05.

**Table 6:** Comparison of blood parameters between premenopausal women with CD and healthy control group.

	Coeliac Group	Healthy Control Group	*p* Value	Reference Value
	*n* = 31	*n* = 39		
Haemoglobin	131.0 ± 10.9 ^†^	130.4 ± 10.3 ^†^	0.7860	<70 g/L severe anaemia ^1^ 80–109 g/L moderate ^1^ 110–119 g/L mild ^1^ >120 g/L non-anaemic ^1^
g/L	(117.0–155.0)	(102.0–156.0)		
	*n* = 27	*n* = 38		
B12	336.0	249.0	0.3477	130–650 pmol/L ^2^
pmol/L	(267.0, 442.5)	(194.5, 373.0)		
Folate	20.3 ± 8.1 ^†^	21.9 ± 8.5 ^†^	0.4073	>8.0 nmol/L ^2^
nmol/L	(9.0–37.0)	(9.0–40.0)		
PTH	3.23 ± 0.92 ^†^	3.42 ± 1.08 ^†^	0.4045	1.6–7.0 pmol/L ^2^
nmol/L	(1.40–5.20)	(1.90–6.10)		
Calcium	2.40	2.40	0.1617	2.2–2.6 mmol/L ^2^
mmol/L	(2.30, 2.40)	(2.30, 2.40)		
CTX-I	0.44 ± 0.17 ^†^	0.51 ± 0.22 ^†^	0.2623	<0.75 µg/L ^2^
µg/L	(0.15–0.74)	(0.17–1.21)		
	*n* = 28	*n* = 38		<25 nmol/L mod-severe deficiency ^2^ 25–50 nmol/L mild Deficiency ^2^ 50–150 nmol/L optimal ^2^
Vitamin D	75.3 ± 22.5 ^†^	80.2 ± 25.4 ^†^	0.4976	
nmol/L	(46.0–130.0)	(38.0–143.0)		

Data presented as mean ± standard deviation ^†^ (Range). Non-parametric data presented as median (25th percentile, 75th percentile). PTH, parathyroid hormone (corrected for albumin); CTX-I, C-terminal telopeptide of type I collagen; vitamin D, 25(OH)D, 25 hydroxyvitamin D. ^1^ Reference values from World Health Organization [37] and ^2^ Canterbury Health Laboratories [38].

**Table 7:** Comparison of physical activity level between premenopausal women with CD (*n* = 30) and healthy control group (*n* = 36).

	Coeliac Group (*n* = 30)	Healthy Control Group (*n* = 36)	*p* Value for Correlation	Recommendation
Moderate Activity Minutes	111 (40, 381)	270 (115, 517.5)	0.1349	150 min
Vigorous Activity Minutes	90 (17.5, 240)	190 (25, 300)	0.4293	75 min
MET Minutes combined	1640 (470, 3190)	2400 (1000, 4980)	0.1622	600 MET minutes

Median (25th percentile, 75th percentile). MET, metabolic equivalent of task.

## Data Availability

The data presented in this study are included in the article, further inquiries can be directed to the corresponding author.
